# Effect of pomegranate extract on blood pressure and anthropometry in adults: a double-blind placebo-controlled randomised clinical trial

**DOI:** 10.1017/jns.2017.36

**Published:** 2017-08-09

**Authors:** A. Stockton, G. Farhat, Gordon J. McDougall, E. A. S Al-Dujaili

**Affiliations:** 1 Department of Dietetics, Nutrition and Biological Sciences, Queen Margaret University, Musselburgh, East Lothian EH21 6UU, UK; 2 Environmental and Biochemical Sciences Group, The James Hutton Institute, Dundee DD2 5DA, UK; 3 Faculty of Pharmacy, Middle East University, Amman, Jordan

**Keywords:** Pomegranate extract, Polyphenols, Blood pressure, Anthropometry, Obesity, Cardiovascular risk, DBP, diastolic blood pressure, PE, pomegranate extract, QMU, Queen Margaret University, SBP, systolic blood pressure

## Abstract

Pomegranate (*Punica granatum*), a polyphenol-rich fruit, has been suggested to reduce cardiovascular risk due to its antioxidant properties. Hypertension and obesity are the most preventable cardiovascular risk factors. Few studies on blood pressure and/or body-weight status have been conducted in human subjects. Previous investigations have tended to focus on pomegranate juice. The aim of the present study was to investigate the effect of pomegranate extract (PE) on blood pressure and anthropometric measures in adults with no symptomatic disease. A total of fifty-five participants enrolled in a randomised double-blinded placebo-controlled clinical trial where they were assigned to either PE capsules or placebo capsules for 8 weeks. Blood pressure, body weight, waist circumference, waist:hip ratio (WHR) and body composition (lean body mass, body fat) were measured at baseline, week 4 and week 8. Results showed a significant decrease in diastolic blood pressure after 8 weeks (by 2·79 (sd 5·32) mmHg; *P* < 0·05), while the decrease in systolic blood pressure did not reach statistical significance (2·57 (sd 7·4) mmHg; *P* > 0·05). Body fat percentage, lean body mass, waist circumference and WHR did not significantly differ between groups at the end of the intervention. Results suggest that PE may reduce blood pressure and possibly prevent hypertension in the normotensive population. Further large trials are required to elucidate this effect.

CVD currently affects more than 7 million individuals in the UK and is responsible for 26 % of total deaths^(^^1^^)^. High blood pressure is the greatest contributor to CVD worldwide and one of the most common CVD risk factors^(^^2^^)^. Obesity remains a global epidemic and is an independent risk factor for CVD. It is also associated with increased blood pressure, morbidity and mortality risks, and decreased life expectancy^(^^3^^)^. The benefits of lowering blood pressure and reducing obesity for the prevention of CVD are well established. Studies have reported that a reduction in systolic blood pressure (SBP) and/or diastolic blood pressure (DBP) by 5 mmHg is clinically significant as it decreased cardiovascular risk by 20 %^(^^4^^)^, the risk of stroke by 20 %^(^^5^^)^ and mortality risk by 7 %^(^^6^^)^. Moreover, a modest weight loss (5–10 %) can significantly decrease risk factors for diabetes and CVD^(^^7^^,^^8^^)^. Therefore, attenuating obesity and blood pressure could have a potential effect on reducing CVD risk in the population.

In addition to diet and exercise, the study of complementary approaches for the prevention and management of CVD is now emerging. Bioactive components, particularly polyphenols, have been studied for their potential beneficial effects on health. Pomegranate has a high antioxidant capacity due its considerable polyphenol content, particularly tannins, anthocyanins and ellagic acid derivatives^(^^9^^)^. Pomegranate has been reported to reduce inflammation^(^^10^^)^, lipid peroxidation, oxidative stress^(^^11^^)^ and insulin resistance^(^^12^^)^. Some studies have shown that pomegranate juice reduces blood pressure in hypertensive^(^^13^^)^ and normotensive populations^(^^14^^,^^15^^)^. However, another study showed that 3 months of pomegranate juice supplementation did not significantly affect blood pressure in patients with CHD^(^^16^^)^. In addition, Mathew *et al.*^(^^17^^)^ showed that pomegranate extract (PE) suppressed the postprandial increase in SBP following a high-fat meal^(^^17^^)^. A randomised controlled parallel trial including twenty-nine participants also illustrated that 4 weeks of daily PE supplementation reduced SBP (from 120·3 (sd 13·3) to 115·6 (sd 13·1) mmHg; *P* = 0·012), while no significant changes occurred in the control group^(^^18^^)^. In relation to obesity, PE has been reported to decrease body-weight gain in animals who were administered a high-fat diet^(^^18^^–^^22^^)^. However, its effects in humans remain unclear. One study reported that pomegranate juice supplementation for 1 month prevented weight gain and body fat increase in obese humans (*P* < 0·05), while the latter parameters significantly increased in the control group administered juice with no polyphenols (*P* < 0·05)^(^^23^^)^. This research aimed to study the potential preventive effect of pomegranate on cardiovascular risk by exploring the effect of PE on SBP (primary outcome), DBP and body-weight status in an adult normotensive population.

## Experimental methods

This trial was registered in clinicaltrials.gov as NCT02017132 (https://clinicaltrials.gov/ct2/show/NCT02017132).

### Participants

Participants were recruited from the local community between April 2013 and December 2013 through advertising in the Queen Margaret University (QMU) research recruitment digest and by word of mouth. Eligible participants included men and women, aged 18–65 years with a BMI between 18 and 34·9 kg/m^2^. Volunteers answered the pre-assessment questionnaire before they registered for the study to ensure that they did not have any symptomatic disease. Exclusion criteria included taking medication for diabetes, heart, liver or kidney disease; weight loss within 2 months preceding the study; pregnancy; lactation; and allergies to pomegranate. Participants with a regular intake of pomegranate were also excluded.

### Ethics

The study was granted ethical approval by the Divisional Ethics Committee at QMU. The intervention was conducted according to the guidelines laid down in the Declaration of Helsinki^(^^24^^)^. Information sheets were provided to all potential volunteers and written informed consent was obtained prior to participation. All collected data were stored according to the Data Protection Act (1998)^(^^25^^)^.

### Study design and protocol

The study had a double-blinded parallel controlled design where participants were randomised to the daily intake of either one PE capsule or placebo capsule for 8 weeks. Each participant attended the QMU laboratory on three visits (baseline, week 4 and week 8), during which clinical and anthropometric measurements were taken. The randomisation process was conducted by technical staff independent of the study who allocated treatment using an Internet random number-generator site^(^^26^^)^. The numbers produced were used to allocate the pomegranate and placebo capsules, which looked identical. These were placed in sealed, labelled and pre-prepared opaque containers. Participants were asked to maintain their usual diet and exercise regimens throughout the intervention.

For each visit, participants were asked to visit the QMU laboratory during a fasting state (after 8 h of food and drink restriction). For consistency, participants were asked to consume one PE or placebo capsule daily after a meal (the same chosen meal each day) with a glass of water. Weight, height, waist circumference, hip circumference, body composition (body fat and lean body mass) and blood pressure measurements were collected at baseline, week 4 and week 8. BMI and waist:hip ratio were then calculated. Total antioxidant capacity, total polyphenols levels and levels of malonaldehyde were measured as biomarkers of compliance through 24-h urine collections at baseline and week 8. Post-intervention measurements were recorded independently on different sheets, without reference to the initial set of measurements.

### Pomegranate extracts for intervention

**Fig. 1.**
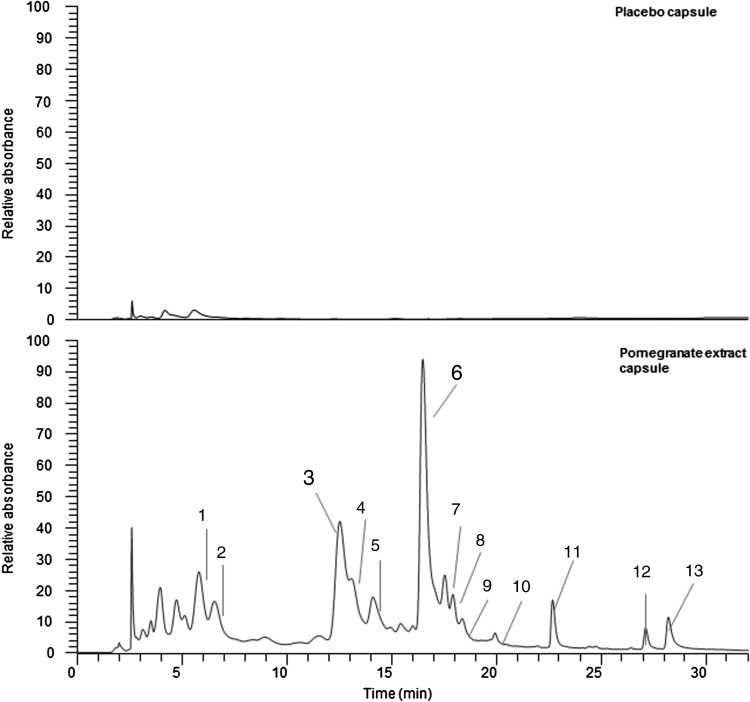
Phenolic content in both placebo and pomegranate capsules. Phenolic components in pomegranate study capsules. UV traces are at 280 nm; placebo and study capsules were extracted as per methods (see Supplementary material). Peaks 3 and 6 are the punicalagin peaks. Full-scale deflection compared at 1·5 × 10^6^ absorbance units to highlight the differences between the samples. Peak identifications are given in Supplementary Table S1.

All pomegranate capsules and placebo capsules were supplied and produced by ProbelteBio in Spain from specially cultivated pomegranates grown on their horticultural farms. Capsules contained a 100 % natural concentrated extract of the whole pomegranate (Pomanox^®^) obtained through a water-based, eco-friendly extraction process. They are standardised for punicalagins. Individual PE capsules comprised: 210 mg of punicalagins, 328 mg of other pomegranate polyphenols (such as flavonoids and ellagic acid) and 0·37 mg of anthocyanins, while placebo capsules consisted of maltodextrin. Both capsules provided a negligible amount of energy (6·52 kcal (27·28 kJ) per capsule). The polyphenol composition of both PE and placebo capsules was validated against the company's data at the James Hutton Institute, Dundee. The LC-MS results confirmed that placebo capsules effectively contained no polyphenols and that the PE capsules contained punicalagins (Fig. 1) at the expected ratio compared with the other polyphenol components (Supplementary material, Supplementary Table S1).

### Blood pressure

Based on the Centers for Disease Control and Prevention (CDC) recommendations, blood pressure was measured by a digital sphygmomanometer (Omron MS-1), with the participants in a seated position, using the right arm, following a 10-min rest. The arm was positioned so that the midpoint of the upper arm was at the same level as the heart, and the cuff was placed 2–3 cm above the pulsation of the brachial artery. Blood pressure was measured three times on each occasion and the average was calculated.

### Anthropometric measures

Anthropometric measurements were conducted following the National Health and Nutrition Examination Survey (NHANES) handbook protocols and methods^(^^27^^)^. Weight was measured in the same clothing, with no shoes, on Salter scales (9018S SV3R). Height was measured on a SECA Leicester stadiometer (no. 5) with a sensitivity of 1 mm. Waist and hip circumferences were measured by a Lufkin W606PM Thinline Executive diameter steel tape yellow clad (6 mm × 2 m; Cooper Tools). Body composition was measured through bioelectrical impedance analysis (BIA) using the Bodystat 1500 (2002) machine. BIA measurements were conducted after the participant had rested in a supine position for approximately 10 min. Participants were fasted and advised to void their bladders before the session.

### Urine collection

A 24-h urine sample collection was required the day before both the baseline and week 8 visits. Total urine was weighed, sampled into 15 ml tubes, then aliquoted for use. Aliquots were frozen at −25°C before testing. Total polyphenols were analysed by the Folin and Ciocalteau method, and the total antioxidant capacity was determined through the ferric-reducing antioxidant power (FRAP) assay. The measurement of malonaldehyde levels (indicator of lipid peroxidation) was performed through the thiobarbituric acid-reactive substances method as previously described^(^^28^^)^.

### Diet diaries

To account for any changes in energy and macronutrient intake (carbohydrate, protein, fat) which could influence the results, a 3 d food diary was collected at baseline and over the same days of the week during week 4. Nutrient intakes were generated using Netwisp Software V4.0 (Tinuviel Software). Food recording support and training, including guidance on portions sizes and household measures, were provided by the researcher to assist participants in completing the food diaries.

### Sample size and statistical analysis

Based on 80 % power, a sample size of fifty-two participants was required to achieve a difference of 5·6 mmHg in SBP between the PE and placebo groups assuming that the common standard deviation is 7 mmHg using a two-group *t* test with a 0·05 two-sided significance level. These assumptions were derived from an exploratory study previously conducted at QMU on PE and blood pressure^(^^18^^)^. Assuming 5 % attrition, fifty-five participants were recruited.

Data were analysed using SPSS for Windows version 21.0 (SPSS) and expressed as means and standard deviations. For multiple comparisons, data were analysed using two-way mixed-model ANOVA with time (baseline, week 4, and week 8) as the within-subject factor, and treatment (PE/placebo) as the between-subject factor. FRAP and total polyphenols in the urine were analysed using ANCOVA. Energy, protein, fat and carbohydrate intakes were analysed using paired *t* tests. Significance was set at *P* ≤ 0·05.

## Results

**Fig. 2.**
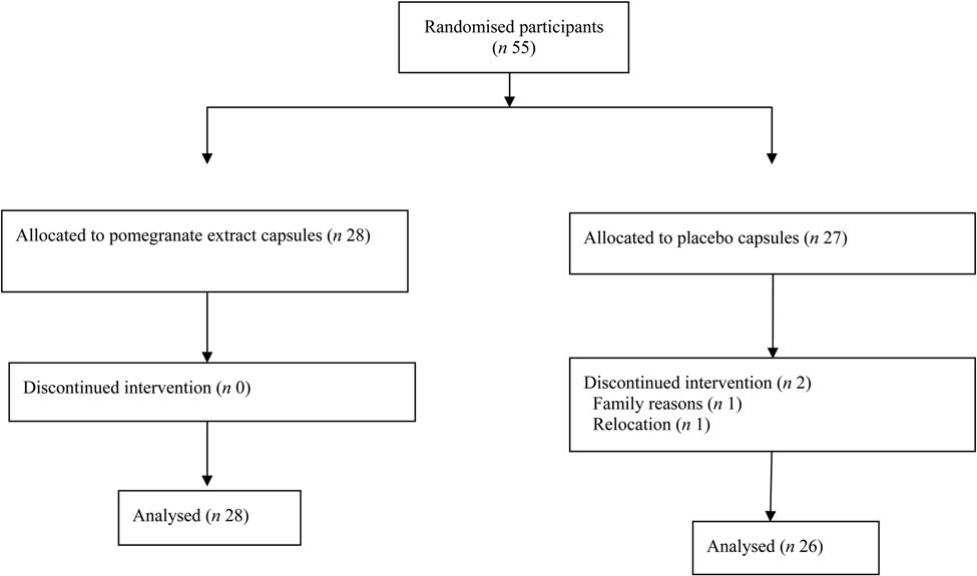
Participant flow diagram.

Of the fifty-five participants in the trial, fifty-three completed the study. There were twenty-two females in the PE group and eighteen in the placebo group. The participant flow diagram is shown in Fig. 2. One participant dropped out before the second appointment and another participant dropped out before the third appointment. Participants with at least two valid time points were included in the analysis. All participants adhered to the protocol. Baseline characteristics of the studied population are shown in Table 1.

**Table 1.** Baseline characteristics of the intention-to-treat dataset (Mean values and standard deviations)

	PE group (*n* 28)		Placebo group (*n* 27)	
	Mean	sd	Mean	sd
Age (years)	30·14	10·95	34·11	11·28
Weight (kg)	70·34	11·3	68·85	12·00
BMI (kg/m^2^)	24·76	3·83	23·57	3·44
WC (cm)	77·25	8·76	76·01	11·02
WHR	0·76	0·54	0·77	0·80
Body fat (%)	27·56	8·56	24·07	7·22
LBM (%)	72·11	8·45	74·78	8·91
SBP (mmHg)	116·46	9·94	116·81	11·81
DBP (mmHg)	71·54	8·57	70·37	7·96

PE, pomegranate extract; WC, waist circumference; WHR, waist:hip ratio; LBM, lean body mass; SBP, systolic blood pressure; DBP, diastolic blood pressure.

### Changes in blood pressure

**Fig. 3.**
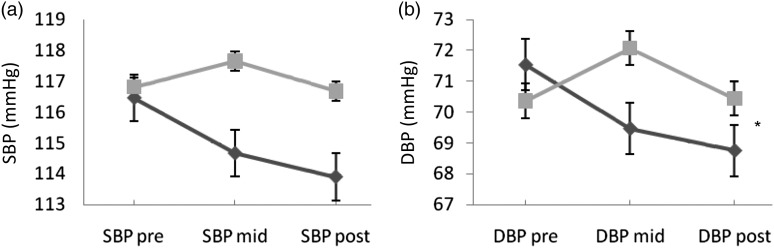
Changes in systolic blood pressure (SBP) (a) and diastolic blood pressure (DBP) (b) between groups at different time points. Results are means, with standard errors represented by vertical bars. * There was a significant interaction between treatment and time for DBP (*F*_2,102_ = 4·4; *P* = 0·02) but not for SBP (*F*_2,102_ = 1·2; *P* = 0·30). DBP decreased by 2·79 (sd 5·32) mmHg after 8 weeks in the pomegranate extract group (-♦-) while the decrease in SBP (by 2·57 (sd 7·4) mmHg) did not reach statistical significance. 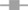, Placebo group.

ANOVA showed no significant interaction between treatment and time for SBP (*F*_2,102_ = 1·2; *P* = 0·30). However, the interaction was significant for DBP (*F*_2,102_ = 4·4; *P* = 0·02). In the PE group, DBP decreased by 2·79 (sd 5·32) mmHg after 8 weeks, while the decrease in SBP (by 2·57 (sd 7·4) mmHg) did not reach statistical significance (Fig. 3).

### Anthropometric measures

Results showed no statistically significant interaction between the intervention and time for body weight (*F*_1·6,81·7_ = 0·24; *P* = 0·74), BMI (*F*_1·7,86·9_ = 0·57; *P* = 0·54), waist circumference (*F*_2,106_ = 0·25; *P* = 0·78), waist:hip ratio (*F*_1·6,86·6_ = 1·08; *P* = 0·34), body fat percentage (*F*_1·5,76·5_ = 2·02; *P* = 0·15) and lean body mass percentage (*F*_1·2,60·3_ = 0·05; *P* = 0·87). In addition, there was no significant difference between the two groups for all the measures (*P* > 0·05). Data are presented in Table 2.

**Table 2.** Changes in outcomes variables at different time points in the pomegranate extract (PE) and placebo groups* (Mean differences and standard deviations)

	PE group (*n* 28)				Placebo group (*n* 26)			
	Difference at week 4 from baseline		Difference at week 8 from baseline		Difference at week 4 from baseline		Difference at week 8 from baseline	
	Mean	sd	Mean	sd	Mean	sd	Mean	sd
Body weight (kg)	0·32	1·1	−0·14	1·43	0·23	1·50	0·3	1·87
BMI (kg/m^2^)	0·11	0·44	0·01	5·46	0·12	0·50	0·15	6·38
WC (cm)	0·17	0·4	0·14	0·41	0·17	0·45	0·21	0·55
WHR	0·00	0·4	0·00	0·14	−0·03	0·14	−0·45	0·20
Body fat (%)	−0·19	2·1	0·45	2·68	0·88	2·40	0·49	2·35
LBM (%)	0·51	3·01	0·68	3·23	0·29	4·36	0·75	4·37

WC, waist circumference; WHR, waist:hip ratio; LBM, lean body mass.

* Data were analysed using ANOVA.

### Total polyphenols and total antioxidant capacity and malonaldehyde levels

After adjustment for pre-intervention levels of all biomarkers, there was no statistically significant difference in post-intervention levels in both groups for total antioxidant capacity (*F*_1,47_ = 0·19; *P* = 0·7), total polyphenol levels (*F*_1,48_ = 0·98; *P* = 0·98) and levels of malonaldehyde (*F*_1,47_ = 0·11; *P* = 0·74) in the urine (Table 3).

**Table 3.** Total antioxidant capacity, total polyphenols and malonaldehyde (MDA) levels in the urine* (Mean values and standard deviations)

	PE group				Placebo group			
	Baseline		Week 8		Baseline		Week 8	
	Mean	sd	Mean	sd	Mean	sd	Mean	sd
TAC (mg GAE/d)	6·86	1·7	6·43	1·86	6·6	1·92	6·49	1·94
Total polyphenols (mg GAE/d)	687·6	239	682	252·6	790	395	676	239
MDA levels (μmol MDA/d)	0·72	0·29	0·75	0·39	0·89	0·39	0·78	0·39

TAC, total antioxidant capacity; GAE, gallic acid equivalents.

* Data were analysed using ANCOVA.

### Diet diaries

Analysis showed no significant differences in baseline levels of energy (*P* = 0·08), carbohydrate (*P* = 0·4), protein (*P* = 0·23) and fat (*P* = 0·06) levels between the PE group and the placebo group. Also, analysis via paired *t* tests showed no significant differences in the energy and macronutrient intake between baseline and week 4 in both groups (Table 4).

**Table 4.** Energy and macronutrient intakes at baseline and week 4 in the pomegranate extract (PE) and placebo groups (Mean values and standard deviations)

			Run-in period		Week 4	
	Intervention	*n*	Mean	sd	Mean	sd
Energy						
kJ	PE	28	8661	1929	8644	3125
	Placebo	26	9452	1766	9242	2096
kcal	PE	28	2070	461	2066	747
	Placebo	26	2259	422	2209	501
Carbohydrate (g)	PE	28	264	98	264	134
	Placebo	26	281	69	280	92
Protein (g)	PE	28	79	19	78	21
	Placebo	26	85	20	80	19
Fat (g)	PE	28	79	28	82	35
	Placebo	26	90	22	87	21

* Data were analysed using the paired *t* test.

## Discussion

This study investigated the effect of 8 weeks of PE supplementation on blood pressure and anthropometric measures in volunteers with no symptomatic disease. Results indicate that PE induced a significant decrease in DBP (by 2·8 (sd 5·32) mmHg) in the PE group and therefore can offer a protection against CVD. Although SBP decreased in the PE group (by 2·57 (sd 7·4) mmHg) compared with the placebo group (by 0·12 (sd 6·02) mmHg) at the end of the intervention, the difference between the groups did not reach statistical significance. The decrease in blood pressure is in line with our previous exploratory study, yet this earlier investigation noted a significant decrease only in SBP but not DBP^(^^18^^)^. The blood pressure-lowering effect is also consistent with previous studies carried out on pomegranate juice. In fact, a recent meta-analysis has established an association between blood pressure and pomegranate juice consumption^(^^29^^)^. Our study presents therefore a novel aspect as limited research has assessed the effect of PE on CVD risk factors. Given the concerns about obesity resulting from a positive energy imbalance^(^^30^^)^, demonstrating beneficial effects of a low-energy extract of pomegranate would be useful. It would overcome the increase in energy intake resulting from consumption of large quantities of juice.

It is worth mentioning that Seeram *et al.*^(^^31^^)^ reported no significant difference in the bioavailability of polyphenols from pomegranate juice (875 mg of polyphenols) and extracts (755 mg of polyphenols). This was demonstrated by similar changes in serum ellagic acid levels following consumption of both pomegranate products on different days^(^^31^^)^. This finding provides a justification for the comparison between different forms of pomegranate. Therefore, it could be suggested that the consistent results obtained with pomegranate juice rely in the use of higher doses of polyphenols compared with our study (PE contained 538 mg of polyphenols). For instance, the study of Tsang *et al.*^(^^15^^)^ reported a significant decrease in both SBP and DBP following the administration of pomegranate juice (842·5 mg of polyphenols) daily for 4 weeks^(^^15^^)^. This provides a rationale for carrying out studies investigating different doses of polyphenols in a PE on blood pressure.

Mechanisms of action of PE on blood pressure may involve the role of polyphenols in reducing serum angiotensin-converting enzyme (ACE) activity as shown in the study of Aviram & Dornfield^(^^13^^)^. The latter studied the effect of 2 weeks of pomegranate juice supplementation on blood pressure and showed a 36 % decrement in serum ACE activity and a 5 % reduction in SBP (*P* < 0·05). The reduction in ACE levels could be related to the antioxidant properties of pomegranate and their beneficial effect on endothelial function and blood pressure^(^^13^^)^. Another potential mechanism involves the role of polyphenols in increasing nitric oxide levels^(^^32^^)^. To clarify which mechanisms may be responsible for these effects requires further human studies.

Importantly, although the significant decrease in DBP was statistically significant, there is a question about the clinical relevance of this finding. A 5 mmHg decrease in DBP was considered to be clinically significant^(^^33^^)^. Therefore, results might not be clinically significant for all participants. This might be explained by the fact that our population was normotensive. For instance, SBP decreased by 5 % in hypertensive participants administered pomegranate juice^(^^13^^)^. Further research is needed to study the clinical effect of pomegranate and bioactive components on blood pressure and demonstrate whether their preventive effects on hypertension can be relevant only in conjunction with other lifestyle medications such as diet and physical activity.

The decrease in DBP was not associated with a significant increase in total polyphenols, total antioxidant capacity or a decrease in malonaldehyde levels in the urine in the PE group compared with the placebo group. Apart from possible non-compliance with the intervention (or the urine collection itself), these outcomes could also be attributed to the intra- and inter-individual variability in the metabolism of polyphenols^(^^34^^)^. Furthermore, despite the importance of determining compliance to polyphenol intervention, the short half-life of polyphenols (1–5 d) can present a potential limitation, even with repeated daily intakes^(^^35^^)^. Therefore, the biomarker of compliance testing in studies with duration of more than 5 d may be affected. There were also marked differences in the volume of the collected 24-h urine between the pre- and post-samples for thirteen participants, indicating a potential decrease in compliance in providing the 24-h samples.

The analysis of body weight, waist circumference and waist:hip ratio did not report significant interaction between time and treatment on the effects. Body composition measurement was undertaken to explain any potential change in body weight, waist circumference and waist:hip ratio resulting from the intervention. This finding is not in line with several animal studies which demonstrated a significant lowering effect of pomegranate on body weight^(^^19^^–^^22^^)^; however, these studies administered PE in the context of a high-fat diet. It has previously been hypothesised that polyphenols might have a counteracting effect on the increase in body weight caused by an increased energy intake^(^^36^^)^. This has been suggested to be mediated by suppressing food intake and inhibiting pancreatic activity^(^^18^^)^. The differing results in this study could be due to the inclusion of normal-weight participants and overweight participants following their usual diet. These findings may then provide a direction for future research in studying the effects of pomegranate in the obese population. Comparing the effect of pomegranate in the context of a normoenergetic and a high-energy diet or high-fat diet would contribute towards testing the hypothesis generated by animal studies.

The study poses both potential strengths and limitations. Three biomarkers were used to assess participant compliance. In addition, the study was randomised and double-blinded which avoided expectation and selection bias. However, the sample size did not provide enough power to explore the effect of other factors such as age and weight status and women represented the majority of the population, which might affect generalisation of results. The findings are also limited by the lack of knowledge of the physiological mechanisms underlying the decrease in DBP.

### Conclusion

This study showed that a PE significantly lowered DBP over 8 weeks and could contribute to the prevention of CVD risk factors. However, its effect on SBP and body-weight status was not significant. Further long-term studies with a larger population and using different doses of polyphenols are required to corroborate these effects and to provide direction for future recommendations.

## Supplementary material

For supplementary material accompanying this paper visit http://dx.doi.org/10.1017/jns.2017.36. click here to view supplementary material

## References

[1] British Heart Foundation (2017) Heart Statistics. https://www.bhf.org.uk/research/heart-statistics (accessed February 2017).

[2] Lopez AD (editor) (2006) Global Burden of Disease and Risk Factors. Washington, DC: World Bank Publications.

[3] Poirier P, Giles TD, Bray GA, (2006) Obesity and cardiovascular disease: pathophysiology, evaluation, and effect of weight loss. Circulation 14, 898–918. 10.1161/CIRCULATIONAHA.106.171016 16380542

[4] Glynn RJ, Gilbert JL, Sesso HD, (2002) Development of predictive models for long-term cardiovascular risk associated with systolic and diastolic blood pressure. Hypertension 39, 105–110. 11799087 10.1161/hy1201.097199

[5] McInnes GT (2005) Lowering blood pressure for cardiovascular risk reduction. J Hypertens Suppl 23, S3–S8. 10.1097/01.hjh.0000165622.34192.fd 15821449

[6] Whelton PK, He J, Appel LJ, (2002) Primary prevention of hypertension: clinical and public health advisory from The National High Blood Pressure Education Program. JAMA 288, 1882–1888. 12377087 10.1001/jama.288.15.1882

[7] Resnick HE, Valsania P, Halter JB, (2000) Relation of weight gain and weight loss on subsequent diabetes risk in overweight adults. J Epidemiol Community Health 54, 596–602. 10890871 10.1136/jech.54.8.596 PMC1731720

[8] Wing RR, Lang W, Wadden TA, (2011) Benefits of modest weight loss in improving cardiovascular risk factors in overweight and obese individuals with type 2 diabetes. Diabetes Care 34, 1481–1486. 21593294 10.2337/dc10-2415 PMC3120182

[9] Gil MI, Tomás-Barberán FA, Hess-Pierce B, (2000) Antioxidant activity of pomegranate juice and its relationship with phenolic composition and processing. J Agric Food Chem 48, 4581–4589. 11052704 10.1021/jf000404a

[10] Kaplan M, Hayek T, Raz A, (2001) Pomegranate juice supplementation to atherosclerotic mice reduces macrophage lipid peroxidation, cellular cholesterol accumulation and development of atherosclerosis. J Nutr 131, 2082–2089. 11481398 10.1093/jn/131.8.2082

[11] Aviram M, Volkova N, Coleman R, (2008) Pomegranate phenolics from the peels, arils, and flowers are antiatherogenic: studies *in vivo* in atherosclerotic apolipoprotein E-deficient (E0) mice and *in vitro* in cultured macrophages and lipoproteins. J Agric Food Chem 56, 1148–1157. 18173244 10.1021/jf071811q

[12] McFarlin BK, Strohacker KA & Kueht ML (2009) Pomegranate seed oil consumption during a period of high-fat feeding reduces weight gain and reduces type 2 diabetes risk in CD-1 mice. Br J Nutr 102, 54–59. 19079947 10.1017/S0007114508159001

[13] Aviram M & Dornfeld L (2001) Pomegranate juice consumption inhibits serum angiotensin converting enzyme activity and reduces systolic blood pressure. Atherosclerosis 158, 195–198. 11500191 10.1016/s0021-9150(01)00412-9

[14] Lynn A, Hamadeh H, Leung WC, (2012) Effects of pomegranate juice supplementation on pulse wave velocity and blood pressure in healthy young and middle-aged men and women. Plant Foods Hum Nutr 67, 309–314. 22648092 10.1007/s11130-012-0295-z

[15] Tsang C, Smail NF, Almoosawi S, (2012) Intake of polyphenol-rich pomegranate pure juice influences urinary glucocorticoids, blood pressure and homeostasis model assessment of insulin resistance in human volunteers. J Nutr Sci 1, e9. 25191556 10.1017/jns.2012.10 PMC4153032

[16] Sumner MD, Elliott-Eller M, Weidner G, (2005) Effects of pomegranate juice consumption on myocardial perfusion in patients with coronary heart disease. Am J Cardiol 96, 810–814. 16169367 10.1016/j.amjcard.2005.05.026

[17] Mathew AS, Capel-Williams GM, Berry SE, (2012) Acute effects of pomegranate extract on postprandial lipaemia, vascular function and blood pressure. Plant Food Hum Nutr 67, 351–357. 10.1007/s11130-012-0318-9 23093401

[18] Stockton A, Al-Dujaili EA, McDougall G, (2015) Effect of pomegranate extract consumption on cardiovascular disease risk factors, stress hormones, and quality of life in human volunteers: an exploratory randomised, double-blind, placebo-controlled trial. EC Nutr 2, 396–411.

[19] Cerdá B, Llorach R, Cerón JJ, (2003) Evaluation of the bioavailability and metabolism in the rat of punicalagin, an antioxidant polyphenol from pomegranate juice. Eur J Nutr 42, 18–28. 12594538 10.1007/s00394-003-0396-4

[20] Lei F, Zhang XN, Wang W, (2007) Evidence of anti-obesity effects of the pomegranate leaf extract in high-fat diet induced obese mice. Int J Obes (Lond) 31, 1023–1029. 17299386 10.1038/sj.ijo.0803502

[21] Zhang L, Gao Y, Zhang Y, (2010) Changes in bioactive compounds and antioxidant activities in pomegranate leaves. Sci Hortic 123, 543–546.

[22] Vroegrijk IO, van Diepen JA, van den Berg S, (2011) Pomegranate seed oil, a rich source of punicic acid, prevents diet-induced obesity and insulin resistance in mice. Food Chem Toxicol 49, 1426–1430. 21440024 10.1016/j.fct.2011.03.037

[23] González-Ortiz M, Martínez-Abundis E, Espinel-Bermúdez MC, (2011) Effect of pomegranate juice on insulin secretion and sensitivity in patients with obesity. Ann Nutr Metab 58, 220–223. 21811060 10.1159/000330116

[24] World Medical Association (2013) Declaration of Helsinki – Ethical Principles for Medical Research Involving Human Subjects. http://www.wma.net/en/30publications/10policies/b3/index.html (accessed March 2017). 10.1001/jama.2013.281053 24141714

[25] National Archives (1998) Data Protection Act 1998. http://www.legislation.gov.uk/ukpga/1998/29/contents (accessed April 2013).

[26] Randomizer (2017) Research randomizer. https://www.randomizer.org/ (accessed January 2017).

[27] CDC (2007) Anthropometry Procedures Manual. https://www.cdc.gov/nchs/data/nhanes/nhanes_07_08/manual_an.pdf (accessed March 2014).

[28] Buege JA & Aust SD (1978) Microsomal lipid peroxidation. Methods Enzymol 52, 302–310. 672633 10.1016/s0076-6879(78)52032-6

[29] Sahebkar A, Ferri C, Giorgini P, (2017) Effects of pomegranate juice on blood pressure: a systematic review and meta-analysis of randomized controlled trials. Pharmacol Res 115, 149–161. 27888156 10.1016/j.phrs.2016.11.018

[30] Krzysztoszek J, Wierzejska E & Zielińska A (2015) Obesity. An analysis of epidemiological and prognostic research. Arch Med Sci 11, 24–33. 25861287 10.5114/aoms.2013.37343 PMC4379361

[31] Seeram NP, Zhang Y, McKeever R, (2008) Pomegranate juice and extracts provide similar levels of plasma and urinary ellagitannin metabolites in human subjects. J Med Food 11, 390–394. 18598186 10.1089/jmf.2007.650 PMC3196216

[32] Basu A & Penugonda K (2009) Pomegranate juice: a heart-healthy fruit juice. Nutr Rev 67, 49–56. 19146506 10.1111/j.1753-4887.2008.00133.x

[33] Law MR, Morris JK & Wald NJ (2009) Use of blood pressure lowering drugs in the prevention of cardiovascular disease: meta-analysis of 147 randomised trials in the context of expectations from prospective epidemiological studies. BMJ 338, b1665. 19454737 10.1136/bmj.b1665 PMC2684577

[34] Santos-Buelga C, Escribano-Bailon MT, Lattanzio V (editors) (2010) Recent Advances in Polyphenol Research, vol. 2 Chichester: Wiley-Blackwell.

[35] Pérez-Jiménez J, Hubert J, Hooper L, (2010) Urinary metabolites as biomarkers of polyphenol intake in humans: a systematic review. Am J Clin Nutr 92, 801–809. 20810980 10.3945/ajcn.2010.29924

[36] Farhat G, Drummond S, Fyfe L, (2015) Comparison of the effects of high *versus* low-polyphenol dark chocolate on body weight and biochemical markers: a randomized trial. EC Nutr 2, 354–364.

