# Supplementary material for: Effect of pomegranate extract on blood pressure and anthropometry in adults: a double-blind placebo-controlled randomised clinical trial
Source: J Nutr Sci. 2017 Aug 9;6:e39. doi: 10.1017/jns.2017.36 (PMC5672313; doi:10.1017/jns.2017.36)
Supplement: Supplementary file 1 [file S2048679017000362sup001.docx]

**Table S1**. Putative Identifications of phenolic compounds in PE capsule based on LC-MS data

| **Peak** | ***m/z* [M-H]** | **MS^2^** | **Identification** |
| --- | --- | --- | --- |
| 1 | 1415***** | 1113, 933, 783, 781, 633 | di(HHDP galloyl glucose) pentose |
| 2 | 783 | 481, 301, 275 | Pendunculagin isomer |
| 3 | 1083 | 781, 721, 601, 575 | α-Punicalagin |
| 4 | 951 | 907, 783, 301 | Granatin B isomer 1 |
| 5 | 951 | 907, 783, 301 | Granatin B isomer 2 |
| 6 | 1083 | 781, 721, 601, 575 | β-Punicalagin |
| 7 | 1085 | 785, 631, 451 | Digalloyl gallagyl hexoside |
| 8 | 935 | 633, 301 | Galloyl diHHDP hexoside |
| 9 | 1567 | N/A | Sanguiin H10 isomer |
| 10 | 633 | 463, 301 | Galloyl HHDP hexoside |
| 11 | 463 | 301 | EA hexoside |
| 12 | 447 | 301 | EA rhamnoside |
| 13 | 301 | N/A | Ellagic acid (EA) |

***** Possible punicalagin isomer in this peak; underlined = major MS^2^ fragments, N/A = MS^2^ not available. HHDP = hexahydroxydiphenoyl unit

Extraction of pomegranate sample capsules for polyphenol analysis

POMANOX® and placebo capsules were extracted in triplicate with 10 mL of 50 % acetonitrile in ultrapure water containing 0.2 % formic acid, vortex mixed to ensure dissolution and placed on a blood rotator at 45 rpm for 30 mins at 4 ºC. After centrifugation (2780 X g, 5 mins, 5 ºC), the supernatants were removed to fresh tubes and the extraction repeated on the pellet. The two extractions were combined (20 mL), mixed and 1 mL aliquots removed and dried in a centrifugal evaporator. The dried samples were re-dissolved in 500 μL of 5 % acetonitrile in ultrapure water containing 0.1 % formic acid.

Liquid Chromatography Mass Spectrometry (LC-MS),

Samples were analysed on an LCQ-Deca system, comprising Surveyor auto-sampler, pump and photodiode array detector (PDAD) and an ion-trap mass spectrometer (Thermo Fisher Scientific, Hemel Hempstead, UK). The PDAD scanned discrete channels at 280 nm, 365 nm and 520 nm. The samples were applied to a C18 column (Synergi Hydro C18, 2 mm X 150 mm, Phenomenex Ltd.) and eluted using a gradient of 5% acetonitrile containing 0.1% formic acid to 40% acetonitrile containing 0.1% formic acid over 30 min at a rate of 200 μL/min. The LCQ-Deca LC–MS was fitted with an ESI (electrospray ionisation) interface and analysed the samples in positive and negative ion mode. All data shown is in negative mode. There were two scan events; full scan analysis followed by data-dependent MS/MS of the most intense ions using collision energies (source voltage) of 45%. The capillary temp was set at 250ºC, with sheath gas at 60 psi and auxiliary gas at 15 psi. Pomegranate components were identified by comparison with existing data.
